# Predictive value of lesion morphology in rectal cancer based on MRI before surgery

**DOI:** 10.1186/s12876-023-02910-4

**Published:** 2023-09-19

**Authors:** Baohua Lv, Xiaojuan Cheng, Yuanzhong Xie, Yanling Cheng, Zhenghan Yang, Zhenchang Wang, Erhu Jin

**Affiliations:** 1 Department of Radiology, Beijing Friendship Hospital, Capital Medical University, No. 95, Yong-an Road, Beijing, 100050 China; 2 Department of Radiology, Taian City Central Hospital, Tai’an, 271099 China; 3 Clinical Skills Center, Taian City Central Hospital, Tai’an, 271099 China; 4 Respiratory department of Shandong second rehabilitation hospital, Tai’an, 271000 China

**Keywords:** EMVI, Local recurrence, Metastasis, MRI, Rectal cancer

## Abstract

**Objective:**

To explore the relationship of MRI morphology of primary rectal cancer with extramural vascular invasion (EMVI), metastasis and local recurrence.

**Materials and methods:**

This retrospective study included 153 patients with rectal cancer. Imaging factors and histopathological index including nodular projection (NP), cord sign (CS) at primary tumor margin, irregular nodules (IN) of mesorectum, MRI-detected peritoneal reflection invasion (PRI), range of rectal wall invasion (RRWI), patterns and length of tumor growth, maximal extramural depth (EMD), histologically confirmed local node involvement (hLN), MRI T stage, MRI N stage, MRI-detected extramural vascular invasion (mEMVI) and histologically confirmed extramural vascular invasion (hEMVI) were evaluated. Determining the relationship between imaging factors and hEMVI, synchronous metastasis and local recurrence by univariate analysis and multivariable logistic regression, and a nomogram validated internally via Bootstrap self-sampling was constructed based on the latter.

**Results:**

Thirty-eight cases of hEMVI, fourteen cases of synchronous metastasis and ten cases of local recurrence were observed among 52 NP cases. There were 50 cases of mEMVI with moderate consistency with hEMVI (Kappa = 0.614). NP, CS, EMD and mEMVI showed statistically significant differences in the negative and positive groups of hEMVI, synchronous metastasis, and local recurrence. Compared to patients with local mass growth, the rectal tumor with circular infiltration had been found to be at higher risk of synchronous metastasis and local recurrence (*P* < 0.05). NP and IN remained as significant predictors for hEMVI, and mEMVI was a predictor for synchronous metastasis, while PRI and mEMVI were predictors for local recurrences. The nomogram for predicting hEMVI demonstrated a C-index of 0.868, sensitivity of 86.0%, specificity of 79.6%, and accuracy of 81.7%.

**Conclusion:**

NP, CS, IN, large EMD, mEMVI, and circular infiltration are significantly associated with several adverse prognostic indicators. The nomogram based on NP has good predictive performance for preoperative EMVI. mEMVI is a risk factor for synchronous metastasis. PRI and mEMVI are risk factors for local recurrence.

## Introduction

Rectal cancer is the third most common malignant tumor in global morbidity and mortality [1], with local site recurrences and metastasis as the two main causes of death in patients with rectal cancer. After potentially curative surgery, an in-depth pathological assessment of the severity of primary tumor invasion and the existence of lymphatic and distant organ metastasis is the basis for prognosis and adjuvant therapy [2]. However, TNM-based testing may not accurately diagnose all patients, especially those with rectal cancer in earlier stages and negative lymph nodes, while other characteristics of tumors identify patients at increased risk and may benefit from adjuvant treatment. EMVI is mainly detected by pathology and high-resolution MRIs [3]. hEMVI is determined postoperatively and could not provide helpful information for the management of preoperative treatment strategies. At present, a five-scoring system is adopted for diagnostic criteria of mEMVI, which is primarily used to assess the changes in vascular morphology and signals on high-resolution T2WI (HRT2WI) or contrast-enhanced T1WI (CET1WI) [4–6]. However, MRI detection presented large variance and lower sensitivity (28-62%) [7–9]. Therefore, observation of the extramural vessels of the rectum only is not enough and it is necessary to consider some indirect signs for lesion diagnosis.

In clinical practices, NP and CS at the tumor margin and IN of mesorectum often appear together with metastasis and EMVI [10, 11]. Therefore, it is speculated that certain morphological changes in the primary tumor may indicate the tumor is more aggressive and will produce a poor prognosis. Moreover, there are very few studies about the morphological features and peritoneal reflection invasions.

This study aims to explore the relationship of MRI morphology of primary rectal cancer with EMVI, metastasis and local recurrence.

## Materials and methods

### Patients

**Fig. 1:**
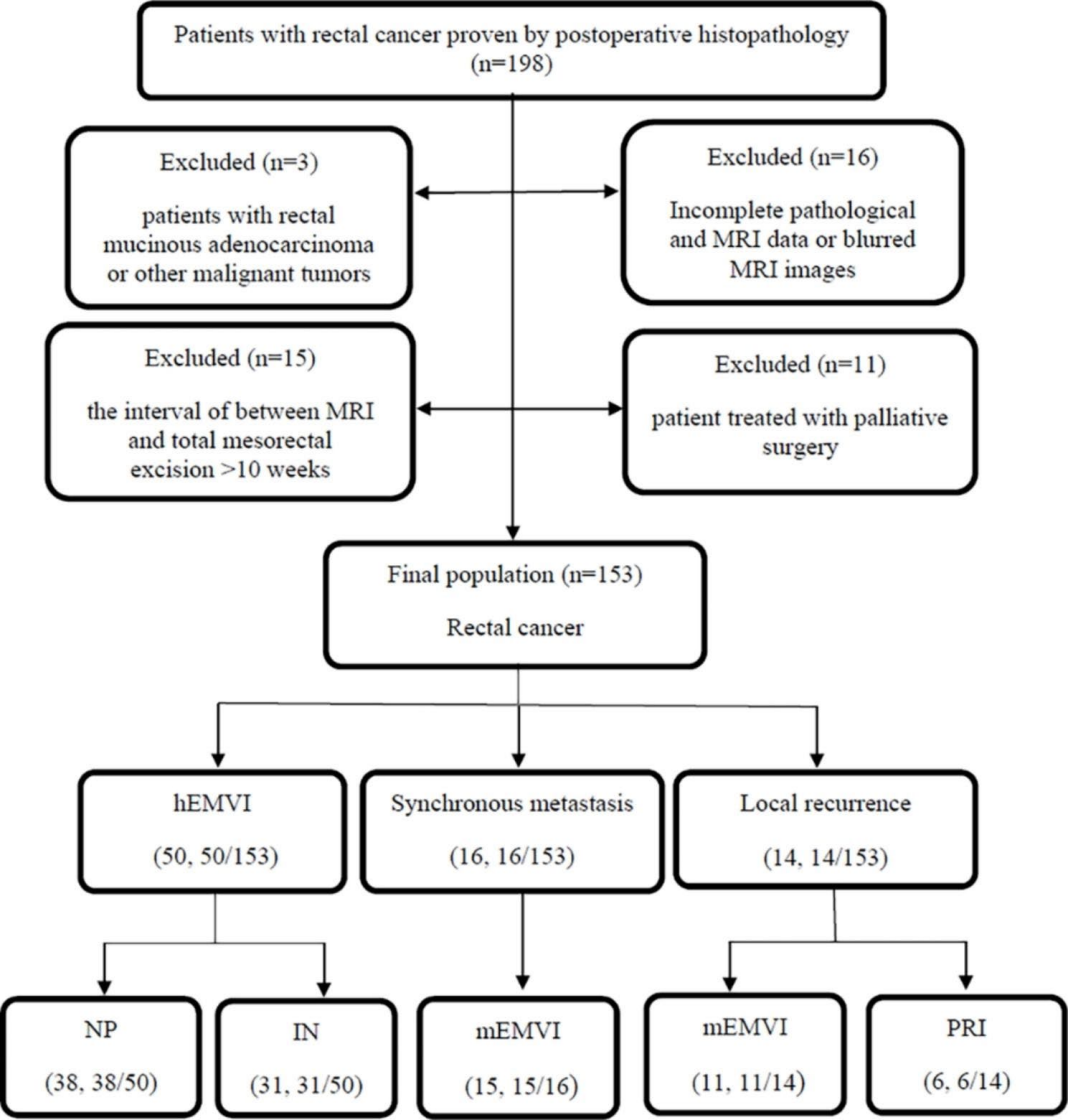
Study flow diagram. hEMVI: histologically confirmed extramural vascular invasion; NP: nodular projection at the primary tumor’s edge; IN: irregular nodules in the mesorectum; mEMVI: MRI-detected extramural vascular invasion; PRI: peritoneal reflection invasion

We conducted a retrospective analysis of medical records and MRI data for 198 patients with rectal cancer who were treated at our hospital between October 2014 and April 2019. All patients were pathologically confirmed to have rectal cancer after surgery. The MRI data collected for this study were obtained prior to surgical intervention or neoadjuvant therapy. The synchronous metastasis information accompanying with rectal cancer was also collected. The exclusion criteria included: (i) patient with rectal mucinous adenocarcinoma because of different biologic behavior; (ii) patient treated with palliative surgery; (iii) patient with incomplete pathological data or MRI data, and patient with blurry images (e.g., motion artifacts); (iv) patient who had a history of other concurrent malignancies; (v) patient who had an interval of more than 10 weeks between MRI and total mesorectal excision. Finally, 153 patients with rectal cancer who underwent total mesorectal excision were included in the study (Fig. 1), of whom 22 patients underwent neoadjuvant therapy prior to surgery. The study was approved by the research committee of our institution and individual consent for this retrospective analysis was waived.

### MRI technique

This specific MRI was conducted by 3.0T system (Signa Excite HD 3.0T, GE Healthcare, Milwaukee, WI, USA) which is configure with a phased-array surface coil. Based on the daily dietary intake, patients were advised to consume light meals within 24 h before the scan and ensure timely bowel movements. Prior to MRI scan, bowel cleansing was performed, and antispasmodic drugs were not administered. Pulse sequences was observed by fast spin-echo sagittal HRT2WI with a thickness of 4 mm, an intersection gap of 1 mm, and a TR/TE of 4,000 ms/102 ms, in absence of fat saturation. The matrix size was 320 × 256. The ETL was 16, and NEX was 4. In addition, an oblique axial (perpendicular to the long axis of the rectum) HRT2WI was performed with a contiguous section thickness of 3 mm, TR/TE of 4,000 ms/102 ms, as well as FOV of 16 × 16 cm without fat saturation. The matrix size was 320 × 256. The ETL and NEX was 16 and 4, respectively. The oblique coronal HRT2WI encompassed both the entire tumor and the anterior and posterior walls of the rectum by being performed parallel to the long axis of the diseased bowel, with a contiguous section thickness of 3 mm, TR/TE of 5800 ms/110 ms, the matrix size of 288 × 288, as well as FOV of 26 × 26 cm without fat saturation.

For CET1WI, a LAVA/LAVA-XV sequence was conducted in the presence of fat saturation, with a thickness of 3 mm, FOV of 36 × 36 cm, a matrix size of 256 × 192 and a flip angle of 15° for 40 consecutive phases. Gd-DTPA (0.1 mmol/kg, Magnevist, Bayer Schering, Germany) was intravenously injected at a rate of 2mL/s, followed by a saline flush prior to enhanced sequencing.

### Morphological data collection

The morphological data of primary tumor were collected by two experienced radiologists from HRT2WI, including NP, CS, IN, PRI, RRWI, growth pattern, tumor length, and EMD. All these images were obtained from the MR findings before the adjunctive therapy. If there is any dispute, a third radiologist with experience in gastrointestinal imaging examination joined the discussion until reaching a final consensus. All the radiologists were informed of the inclusion criteria in this study, and kept blind to the pathological results of EMVI in patients.

mEMVI was determined based on five-scoring system [4]. (i) 0 score: the tumor was confined to the intestinal wall, no nodular protrusion was found at the edge of the tumor, and no vascular development was found near the tumor. (ii) 1 score: nodular protrusions at the tumor margin extended into the mesenteric fat, and there was no vascular development around the tumor. (iii) 2 score: nodular protrusions at the tumor margin extended into the mesentery fat, normal blood vessels were observed beside the tumor, no abnormal blood vessels were observed in CET1WI. (iv) 3 score: HRT2WI showed nodular protrusions at the tumor margin extending into the mesenteric fat, mild dilation of the paratumeric vessels, and tumor signals in the lumen. In CET1WI, the degree of lumen enhancement of the lesion vessels was less than that of the normal vessels, and the abnormal signals similar to tumor enhancement appeared in the lumen, and the lumen was slightly dilated. (v) 4 score: HRT2WI showed nodular protrusions at the tumor margin extending into the mesenteric fat, one or more obviously irregular dilated blood vessels near the tumor, and tumor signals in the lumen. CET1WI showed significant and irregular dilation of lumen of one or more pathological vessels, with less enhancement than normal vessels, and abnormal signals of tumor-like enhancement appeared in lumen. (vi) 3 or 4 score: IN involved one or more adjacent vessels.

**Fig. 2:**
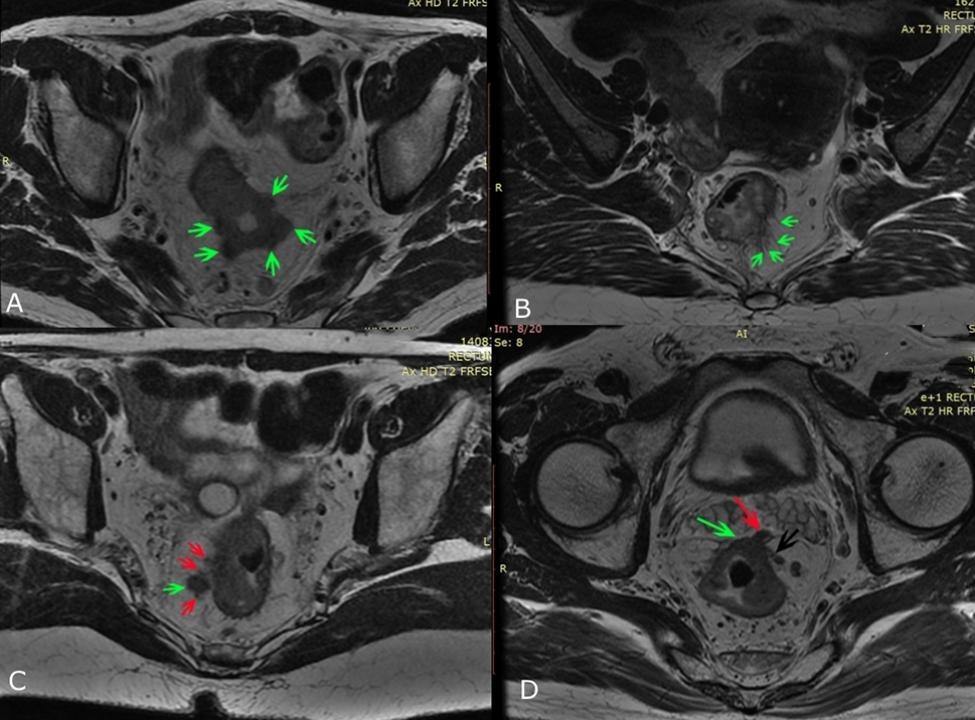
Diagrams of NP, CS, IN and PRI **A**: Multiple NPs (green arrows) appeared at the margin of rectal primary mass **B**: Multiple CSs (green arrows) appeared on the left margin of rectal primary mass **C**: An IN (green arrow) appeared next to the right posterior aspect of the rectal cancer mass, with unsmooth marginal lobing and poor demarcation from the adjacent CS (red arrows) **D**: The peritoneal reflection invaded by the rectal cancer mass was thickened irregularly (green arrow), and the mass breaked through the peritoneal reflection to form NP (red arrow); a vein at the left margin of the rectal cancer mass was invaded (black arrow), the lumen widens and moderate signal tumor tissue appeared in the lumen

**Fig. 3:**
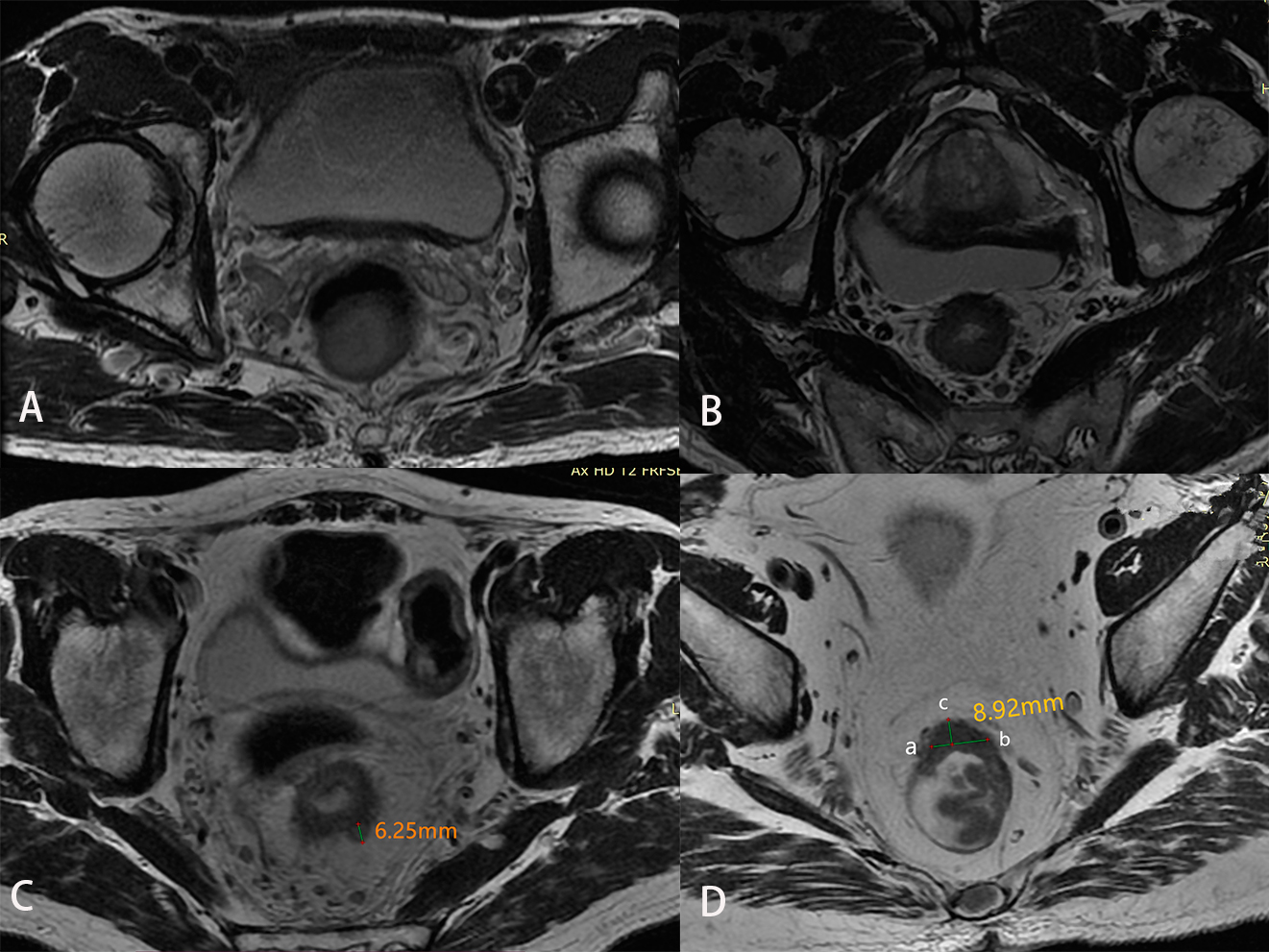
Diagrams of growth pattern and measurement of EMD **A**: A round mass appeared in the posterior rectal wall, protruding into the rectal lumen. **B**: Rectal cancer masses grew in a circular pattern along all rectal walls **C**: The EMD from the tumor farthest edge to the residual muscular layer was approximately 6.25 mm **D**: The remnants of the intrinsic muscular layer at the edge of the rectal cancer mass were marked as points a and b. The vertical distance of 8.92 mm from the farthest point c of the mass to the line connecting points a and b was the EMD

NP is defined as a tumor that breaks through the muscle layer and forms more than one nodule in surrounding adipose tissue (Fig. 2A). CS is a cord that extends from the mass to surrounding adipose tissue, with uneven thickness and hairy edges (Fig. 2B). If CET1WI indicated normally enhanced vessels for this cord, the possibility of CS was excluded. IN is defined as presence of mesenteric nodule with irregular form, rough edge, lobulated appearance, or burrs in HRT2WI (Fig. 2C). PRI means the tumor was indistinctly fused with peritoneal reflection on oblique axial HRT2WI or on sagittal T2WI, or thickening of the peritoneal reflection and signal changes were observed (Fig. 2D). The growth pattern mainly includes local mass and circular infiltration. A local mass means a tumor shows a rounded or oval profile. If the mass is irregular, but is confined to one or two lateral walls, and the overall diameter is wider than one-half of the length, it is also defined as local mass (Fig. 3A). A tumor growing in a circular pattern along the rectal wall is defined as circular infiltration (Fig. 3B). Circular infiltration refers to tumor growth along the intestinal wall on oblique axial HRT2WI, which surpassed one half of the intestinal wall. The tumor morphology was in a circular or semi-circular shape, rather than a round-like or ellipse profile (Fig. 3B).

### Measurement of RRWI, tumor length and EMD

RRWI is measured around the circumference of the intestinal lumen on oblique axial HRT2WI. Tumor length is the distance between the upper quadrant and lower quadrant of the tumor measured on sagittal T2WI along the longitudinal axis of the tumor (multi-dot measurement along the curve of tumor). EMD is the distance from the most distal edge of the tumor to the residual intrinsic muscle layer on oblique axial HRT2WI (Fig. 3C). For unrecognized muscularis propria, the vertical distance from the most distal edge of the tumor to the line connecting two sides of the residual intrinsic muscular layer is measured on oblique axial HRT2WI (Fig. 3D) [11].

### MRI determined T and N stage

The tumor stage assessed by MRI is largely determined by the signal differences in T2 signal intensity in the tumor, submucosa, muscular layer, and mesorectum on short-axial T2WI. T1 is for tumor invades submucosa; T2 for tumor invades muscularis propria; T3 for tumor invades the muscularis propria and pericolorectal tissues; T4 for tumor penetrates the peritoneal surface of the viscera, or invades other organs or structures. MRI-detected metastatic lymph node was confirmed using three conditions [12]: (i) the nodule with a short diameter of more than 1.0 cm; (ii) the nodule with short diameters between 5 and 10 mm were characterized by heterogenous internal signals and irregular or lobulated margins.; (iii) the nodule with short diameters less than 5 was shown to have inhomogeneous internal signals, high signal on high b-value DWI and rough or lobulated at the edges. MRI did not detect any metastatic lymph nodes, which corresponded to N0 stage. The presence of 1–3 metastatic lymph nodes corresponded to N1 stage, while the presence of 4 or more metastatic lymph nodes corresponded to N2 stage.

### Pathological data and follow-up data

Local lymph node involvement and EMVI diagnosis information from histopathological examination were collected and analyzed. EMVI was confirmed by histopathology when the tumor tissue existed within an extramural space or within a tubular structure lined by endothelial cells, smooth muscles, or elastic fibers [13]. Local recurrence data was collected from follow-up data. Follow up period was two years after surgery. During follow up, pelvic MRI, liver ultrasound and serum tumor marker were tested every three months, and colonoscopy every six months. In addition, chest CT and whole abdomen CT were carried out once a year for patients with low rectal cancer, positive circular incisal margin and positive EMVI. Distant metastasis and local recurrence were determined by biopsy or typical imaging presentations.

### Statistical analysis

The statistical package of IBM SPSS Statistics version 26 for Windows was adopted for data analysis. The Shapiro Wilk test was applied to check whether the variables were normally distributed. The continuous variables were compared by T-test, while the variables between groups were classified by the Chi-squared test or Fisher’s exact test. The evaluation of EMVI efficiency was based on the receiver operator characteristic curve (ROC) and area under the curve (AUC). The variables with p < 0.05 in the univariate analysis were included in a binary logistic regression model. In this model, hEMVI, synchronous metastasis and local recurrence were used as dependent variables, while NP, CS and other variables were used as independent variables. The binary logistic stepwise regression (Forward: LR) method was utilized to analyze the impact of NP on hEMVI, synchronous metastasis and local recurrence, after adjusting for confounding factors. Multivariate logistic regression was used to evaluate the variables that are statistically significant in univariate analysis and identify the independent predictors. A nomogram was created using R software (R 4.2.1) based on the results of the multivariable logistic regression analysis, and the C-indices of the nomograms were calculated to determine the differentiation of the models. The comparisons among the ROC curves of the indicators were performed by DeLong test. Interobserver agreement and agreement between pathological findings and imaging assessment results were performed using the Kappa test (Kappa > 0.75 indicated good consistency, 0.40 > Kappa ≤ 0.75 indicated moderate consistency, and Kappa ≤ 0.40 indicated poor consistency). A P value of less than 0.05 indicated a statistical significance.

## Results

### Patient characteristics

A total of 153 (male: 100; female: 53; mean age: 63.75 ± 10.7 yrs; age range: 33–89 yrs) rectal cancer patients admitted to our hospital from October 2014 to April 2019 were included in this study. Demographic data, MRI data and histopathology results were presented in Table 1.

**Table 1:** Results of univariate analysis

	Total	hEMVI			Synchronous metastasis			Local recurrence		
		Positive	Negative	*p* value	Positive	Negative	*p* value	Positive	Negative	*p* value
	153	(n = 50) (%)	(n = 103) (%)		(n = 16) (%)	(n = 137) (%)		(n = 14) (%)	(n = 139) (%)	
Age	63.7 ± 10.7	62.8 ± 11.1	62.8 ± 9.9	0.47	60.1 ± 6.6	64.2 ± 11.0	0.15	60.6 ± 12.5	63.9 ± 10.7	0.42
Gender	153			0.91			0.42			0.49
Male	100(65.4)	33(66.0)	67(65.0)		9(56.3)	91(66.4)		12(73.3)	88(64.5)	
Female	53(34.6)	17 (34.0)	36(35.0)		7(53.7)	46(36.6)		2(26.7)	51(35.5)	
NP				< 0.001			< 0.001			0.003
Negative	101(66.0)	12(24.0)	89(86.4)		2(12.5)	99(72.3)		4(28.6)	97(69.7)	
Positive	52(34.0)	38(76.0)	14(13.6)		14(87.5)	38(27.7)		10(71.4)	42(30.2)	
CS				< 0.001			0.02			0.006
Negative	100(65.4)	15(30.0)	85(82.5)		5(31.2)	95(69.9)		4(28.6)	96(69.1)	
Positive	53 (34.6)	35(70.0)	18(17.5)		11(68.8)	42(30.1)		10(71.4)	43(30.9)	
IN				< 0.001			< 0.001			0.25
Negative	111(72.5)	19(38.0)	92(89.3)		4(25.0)	107(78.1)		9(60.0)	102(73.9)	
Positive	42(27.5)	31(62.0)	11(10.7)		12(75.0)	30(21.9)		5(40.0)	37(26.1)	
PRI				0.02			0.22			< 0.001
Negative	141(92.2)	42(84.0)	99(96.0)		13(81.3)	128(93.4)		8(57.1)	133(95.7)	
Positive	12(7.8)	8(16.0)	4(3.9)		3(18.8)	9(6.6)		6(42.9)	6(4.3)	
Growth pattern				0.38			0.03			< 0.001
Local mass	72(47,1)	21(42.0)	52(50.5)		3(18.8)	69(50.4)		1(7.1)	71(51.1)	
Circular infiltration	81(52.9)	29(58.0)	51(49.5)		13(81.3)	68(49.6)		13(92.9)	68(48.9)	
RRWI				0.48			0.11＊			0.02
≤ 1/3	11(7.2)	2(4.0)	9(8.7)		0(0)	11(8.0)		0(0)	11(7.9)	
1/3 − 2/3	55(35.9)	17(34.0)	38(36.9)		3(18.8)	52 (38.0)		1(7.1)	54(38.8)	
≥ 2/3	87(56.9)	31(62.0)	56(54.4)		13(81.3)	74(54.0)		13(92.9)	74(53.2)	
Tumor length				0.58			0.20			0.06
< 5 cm	90(58.8)	31(62.0)	59(57.3)		7(43.8)	83(60.6)		5(35.7)	85(61.2)	
≥ 5 cm	63(41.2)	19(38.0)	19(42.7)		9(56.2)	54(39.4)		9(64.3)	54(39.8)	
EMD				< 0.001			< 0.001			< 0.001
< 5 mm	99(64.7)	16(32.0)	83(80.6)		3(18.8)	96(70.1)		2(14.3)	97(69.8)	
≥ 5 mm	54(35.3)	34(68.0)	20(19.4)		13(81.3)	41(29.9)		12(85.7)	42(30.2)	
hLN				< 0.001			0.23			0.03
Negative	105(68.6)	20(40.0)	85(82.5)		7(43.7)	98(71.5)		6(42.9)	99(71.2)	
Positive	48(31.4)	30(60.0)	18(17.5)		9(56.3)	39(28.5)		8(57.1)	40(28.8)	
hEMVI							0.01			0.04
Negative	103(67.3)				5(31.2)	98(71.5)		6(42.9)	97(69.7)	
Positive	50(32.7)				11(68.8)	39(28.5)		8(57.1)	42(30.2)	
MRI T stage				0.06			0.02＊			< 0.001＊
T2	23(15.0)	3(6.0)	20(19.4)		0(0)	23(16.8)		0(0)	23(16.5)	
T3	95(62.1)	29(58.0)	66(64.1)		7(43.7)	88(64.2)		5(35.7)	90(64.7)	
T4	35(22.9)	18(36.0)	17(16.5)		9(56.3)	26(19.0)		9(64.3)	26(18.7)	
MRI N stage				< 0.001			< 0.001			0.174
N0	89(58.2)	11(22.0)	78(75.7)		1(6.3)	88(64.2)		5(35.7)	84(60.4)	
N1	33(21.6)	17(34.0)	16(15.5)		4(25.0)	29(21.2)		4(28.6)	29(20.9)	
N2	31(20.3)	22(44.0)	9(8.7)		11(68.8)	20(14.6)		5(35.7)	26(18.7)	
mEMVI				< 0.001			< 0.001			< 0.001
Negative	103(67.3)	13(26.0)	90(87.4)		1(6.2)	102(74.5)		3(21.4)	100(71.9)	
Positive	50(32.7)	37(74.0)	13(12.6)		15(93.8)	35(25.5)		11(78.6)	39(28.1)	

hEMVI: histologically confirmed extramural vascular invasion; NP: nodular projection at the primary tumor’s edge; CS: cord sign at the primary tumor’s edge; IN: irregular nodules in the mesorectum; PRI: peritoneal reflection invasion; RRWI: range of rectal wall invasion; hLN: histologically confirmed local node involvement; EMD: the maximal extramural depth. ＊:Fisher’s exact test; mEMVI: MRI-detected extramural vascular invasion

### Pathological results

Among the 153 patients, 50 (50/153, 32.7%) were confirmed to be hEMVI positive and the other 103(103/153, 67.3%) cases were hEMVI negative according to pathological and immune histochemistry results. Forty-eight (48/153, 31.4%) local lymph node involvements were confirmed according to histological features, thirty (30/48, 62.5%) of which were from hEMVI positive group. There were 26 (26/153, 17%) cases of metastasis, 16 (16/26, 61.5%) of which were synchronous metastasis (8 hepatic metastasis, 5 pulmonary metastasis, 2 hepatopulmonary metastasis and 1 sacral metastasis). Other pathological results were shown in Table 1.

### Results of univariate analysis and ROC

Among the 153 patients, there were 52 (52/153, 34%) cases of NP, 38 (38/50, 76%) of which were from hEMVI positive group and 14 (14/103, 13.6%) of which were from hEMVI negative groups. A total of 50 cases with mEMVI were identified, 37 (74%) of which were from the hEMVI-positive group. mEMVI and hEMVI were moderately consistent, with a Kappa value of 0.614. There is a high degree of agreement between two observers in the evaluation of mEMVI (Kappa = 0.764). Of the 52 cases of NP, 14 (14/52, 27%) and 10 (10/52, 19.2%) had synchronous metastasis and local recurrences, respectively, with significantly higher incidence than the NP-negative group (2/101, 2%; 4/101, 4.0%). According to Table 1, MRI features, such as NP, CS, EMD and mEMVI were significantly associated with hEMVI, synchronous metastasis and local recurrence (P < 0.05). Patients with local lymph node involvement or peritoneal reflection invasion were more likely to develop EMVI and local recurrence. Synchronous metastasis and local recurrence were more likely to occur in tumors with circum wall infiltration. The higher the N stage, the more likely to occur EMVI and synchronous metastasis. The ROC for the evaluation of EMVI, synchronous metastasis and local recurrence diagnostic efficiency for NP was 0.812, 0.799 and 0.706, respectively. The sensitivity and specificity of EMVI for NP and mEMVI were 76.0% vs. 74.0% and 86.4% vs. 87.4%, while those for CS and IN were 70% and 82.5%, as well as 62% and 89.3%, respectively (Table 2). The sensitivity and specificity of synchronous metastasis and local recurrence for NP were in Tables 3 and 4.

**Table 2:** Evaluation of EMVI diagnostic efficiency

	Sensitivity	Specificity	PPV	NPV	Consistent rate	AUC
NP	76.0	86.4	73.1	88.1	83.0	0.812(0.733–0.891)
CS	70.0	82.5	66.0	85.0	78.4	0.763(0.677–0.848)
IN	62.0	89.3	73.8	82.9	80.4	0.757(0.667–0.846)
hLN	60.0	82.5	62.5	81.0	75.2	0.713(0.621–0.805)
Growth pattern	58.0	49.5	35.8	70.8	52.3	0.462(0.365–0.560)
EMD	68.0	80.6	63.0	86.5	76.5	0.743(0.655–0.831)
mEMVI	74.0	87.4	74.0	87.4	83.0	0.807(0.726–0.888)
Nomogram	86.0	79.6	67.2	92.1	81.7	0.868(0.806–0.930)

**Table 3:** Evaluation of synchronous metastasis diagnostic efficiency

	Sensitivity	Specificity	PPV	NPV	Consistent rate	AUC
NP	87.5	72.3	26.9	98.0	73.9	0.799(0.693–0.905)
CS	68.8	69.3	68.8	95.0	69.3	0.690(0.552–0.829)
IN	75.0	78.1	28.6	82.9	96.4	0.766(0.636–0.895)
hLN	56.3	92.5	56.3	93.3	70.0	0.639(0.490–0.788)
EMD	81.3	70.1	24.1	97.0	71.2	0.757(0.636–0.877)
mEMVI	93.8	74.5	30.0	99.0	76.5	0.807(0.726–0.888)

**Table 4:** Evaluation of local recurrence diagnostic efficiency

	Sensitivity	Specificity	PPV	NPV	Consistent rate	AUC
NP	71.4	69.8	9.3	96.0	70.0	0.706 (0.562–0.850)
CS	71.4	69.1	18.9	69.3	69.3	0.702 (0.558–0.847)
IN	40.0	73.9	14.3	91.9	70.6	0.545 (0.383–0.708)
hLN	46.7	70.3	14.6	92.4	68.0	0.642 (0.484–0.800)
EMD	73.3	68.8	20.4	96.0	69.3	0.777 (0.660–0.895)
PRI	33.3	94.9	41.7	92.9	88.9	0.693 (0.520–0.866)
mEMVI	78.6	71.9	9.9	97.1	72.5	0.753 (0.620–0.885)
Nomogram	85.7	70.5	22.6	98.0	71.9	0.827 (0.712–0.943)

### Independent predictors for EMVI, synchronous metastasis and local recurrence

**Fig. 4:**
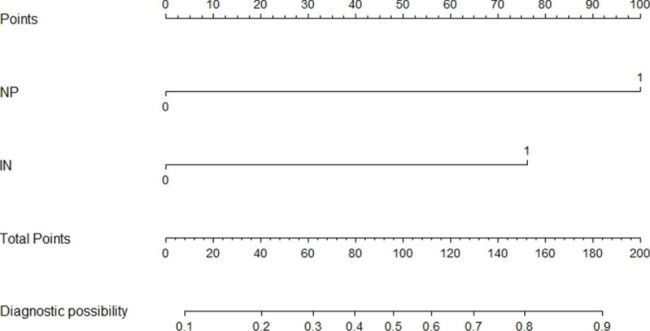
Nomogram of EMVI for IN and NP The risk of developing EMVI of a patient with rectal cancer subjected only to nodular projection (NP) at the primary tumor margin and total points of 100 was approximately 52.5%. The total points were 177 (100 + 77), and the risk of EMVI for this patient was 87.5% if this patient had both NP and irregular nodules (IN) in the mesorectum

**Fig. 5:**
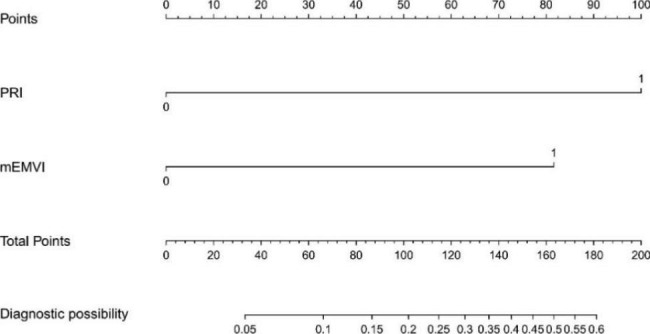
Nomogram of local recurrence for PRI and mEMVI The risk of developing local recurrence of a patient with rectal cancer subjected only to MRI-detected peritoneal reflection invasion (PRI) and total points of 100 was approximately 20%. The total points were 182 (100 + 82), and the risk of local recurrence for this patient was 60% if this patient had both PRI and MRI-detected extramural vascular invasion (mEMVI)

**Fig. 6:**
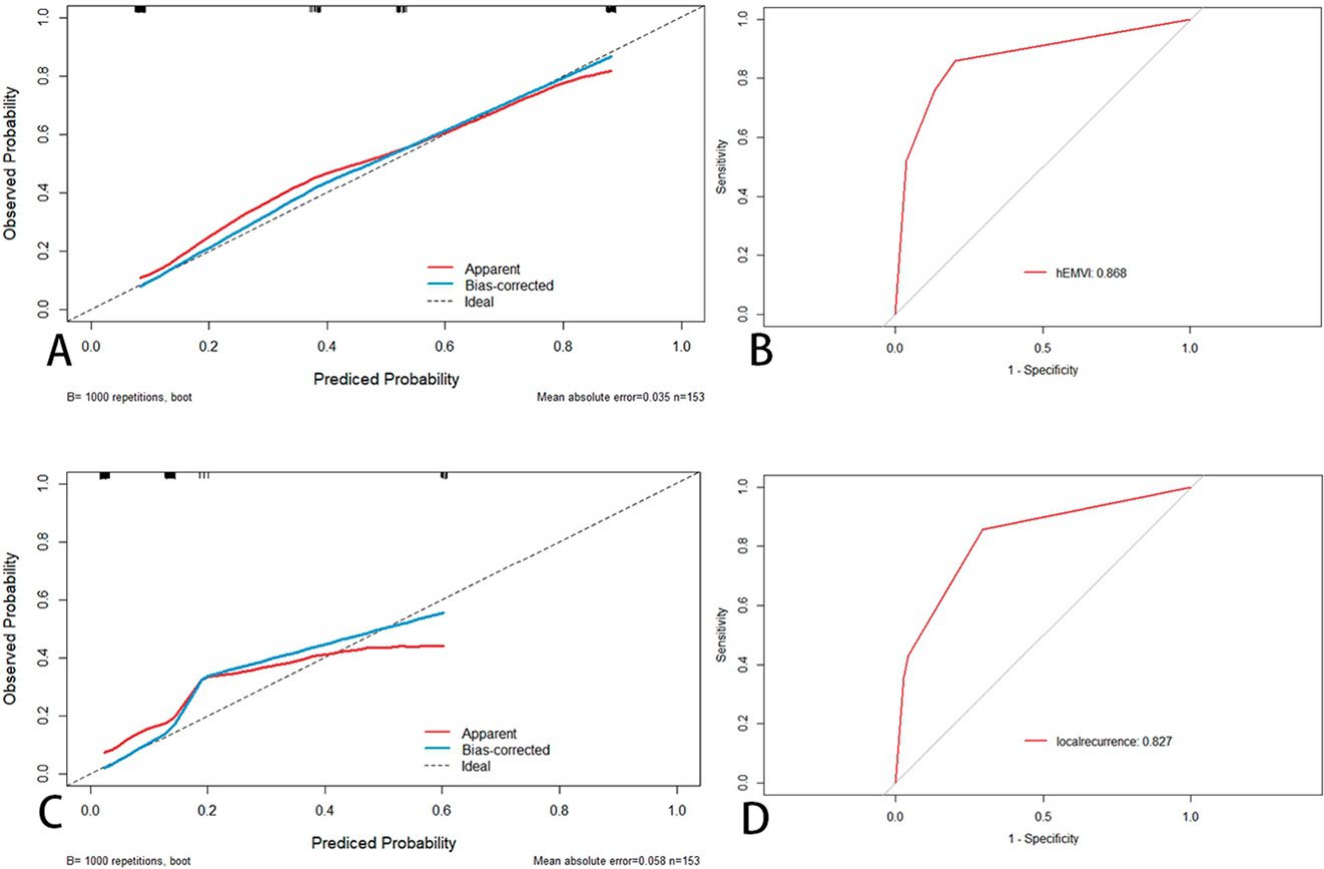
The calibration curve and ROC of the nomogram of EMVI (A + B) and local recurrence (C + D) A + C: the x-axis represents the nomogram-predicted probability and the y-axis represented the actual probability of hEMVI and local recurrence. The perfect prediction corresponded to the 45° dotted line. The red solid line represents the entire cohort (n = 153), and the blue solid line was the bias-corrected value by bootstrapping (B = 1000 repetitions), indicating the observed nomogram performance. The calibration curve showed an evident relationship between the actual tag and the predicted tag. B: the area under the ROC curve of the nomogram for predicting hEMVI was 0.868, suggesting that the confidence level of the probability of EMVI predicted by this nomogram was 86.8%. D: the area under the ROC curve of the nomogram for predicting local recurrence was 0.827, suggesting that the confidence level of the probability of local recurrence predicted by this nomogram was 82.7%

**Fig. 7:**
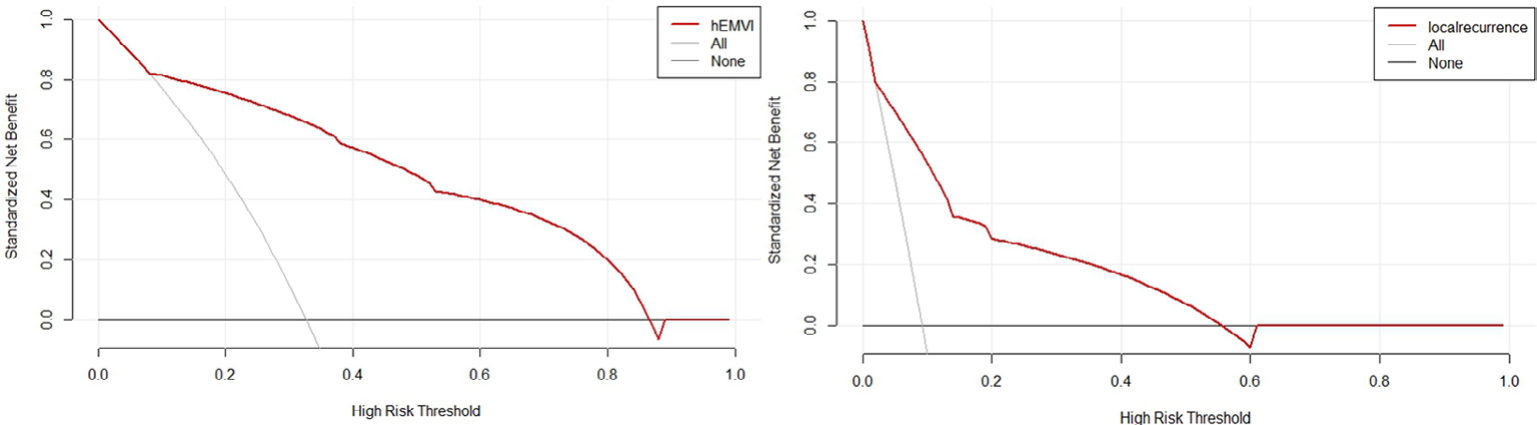
The decision curve analysis (DCA) of the nomogram of hEMVI (**A**) and local recurrence (**B**) **A**: the net benefit of the nomogram prediction model for hEMVI was higher than that of full intervention and no intervention when the prediction threshold was between 0.08 and 0.88, indicating that the column chart model has good clinical applicability. **B**: the net benefit of the nomogram prediction model for local recurrence was higher than that of full intervention and no intervention when the prediction threshold was between 0.02 and 0.58, indicating that the column chart model had a good clinical applicability

**Fig. 8:**
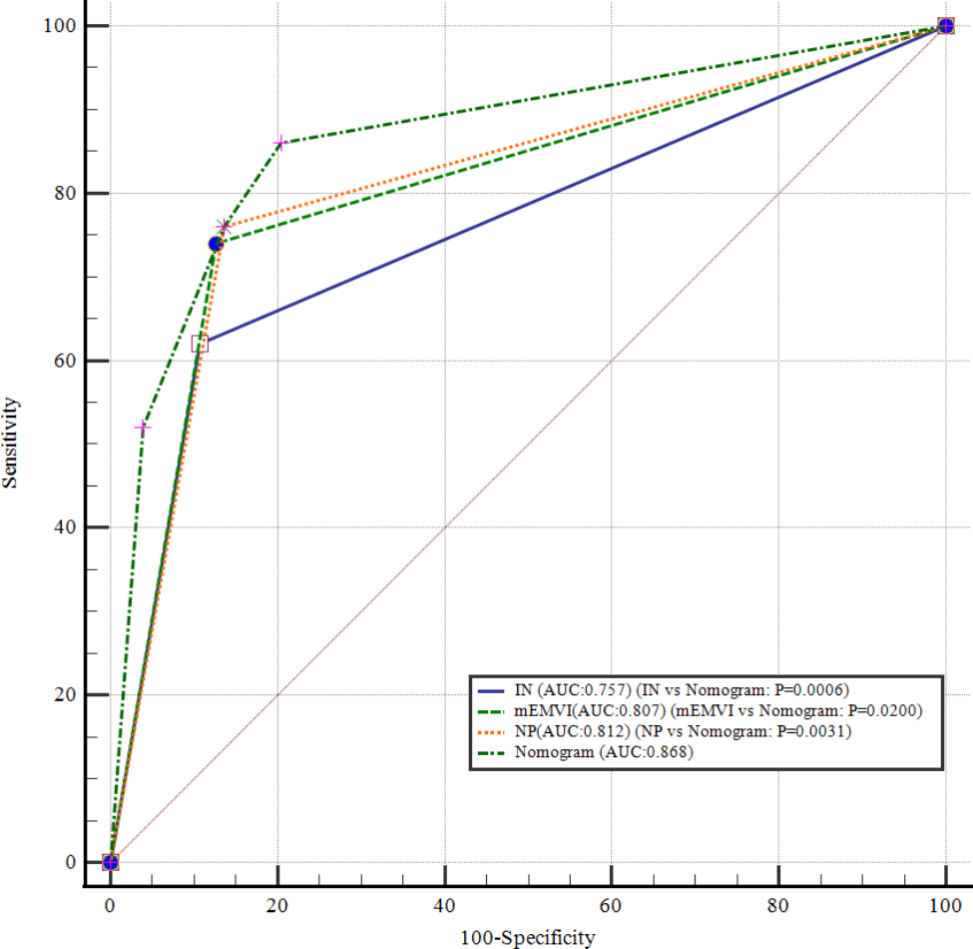
Comparison of the ROC curves of MRI signs and the nomogram The DeLong test showed that the nomogram had a better predictive performance for preoperative EMVI than NP, IN, and mEMVI

All univariately analyzed variables mentioned in Table 1 were entered in the multivariable model. After stepwise removal based on P value, NP and IN remained as significant predictors for hEMVI [OR (95%CI) = 12.1(4.8–30.5), P < 0.001; 6.7(2.5–17.9), P < 0.001], respectively. mEMVI remained as a significant predictor for synchronous metastasis [OR (95%CI) = 43.7(5.8-343.1), P < 0.001]. mEMVI and PRI were independent predictors for local recurrence, with odds ratios of 6.3 (95%CI = 1.6–25.6) and 9.6 (95%CI = 2.3–40.2), respectively (Table 5). Hosmer–lemeshow test shows that the results are not statistically significant, which means that the goodness of fit is good. In addition, we summarized and analyzed the classified risk factors, and analyzed the clinical characteristics with nomograms (Figs. 4 and 5), aiming to provide some guidance for clinicians. The calibration curves analysis (Fig. 6A and C), ROC curves of nomograms (Fig. 6B and D) and decision curve analysis (DCA) (Fig. 7) were plotted, demonstrating the clear correlation between actual and predicted tags, satisfactory discriminative ability and good clinical utility, among them, the nomogram of EMVI had excellent accuracy (Fig. 6A) and differentiation (Fig. 6B, C-index = 0.868). The DeLong test showed that the nomogram had a better predictive performance for preoperative EMVI than mEMVI (P = 0.0200) and NP (P = 0.0031) (Fig. 8).

**Table 5:** Results of multivariable analysis

	hEMVI			synchronous metastasis			local recurrence		
	Odds ratio	95% CI	*p* value	Odds ratio	95% CI	*p* value	Odds ratio	95% CI	*p* value
NP	12.1	4.8–30.5	< 0.001			0.21			0.26
IN	6.7	2.5–17.9	< 0.001			0.27			0.06
PRI			0.73				9.6	2.3–40.2	0.002
mEMVI			0.19	43.7	5.8-343.1	< 0.001	6.3	1.6–25.6	0.010

NP: nodular projection at the primary tumor margin; PRI: peritoneal reflection invasion; IN: irregular nodules in the mesorectum; EMD: the maximal extramural depth. mEMVI: MRI-detected extramural vascular invasion

## Discussion

The rate of distant metastasis from rectal cancer was 25–40% in previous study [14], and the rate of distant metastasis in this study was only 17%, which may be related to the sample difference and the short follow-up time. Liver and lung metastasis are the most common among distant metastasis with a poor prognosis. In this study, we analyzed the correlation between morphological features of rectal primary masses and hEMVI, postoperative local recurrence and synchronous distant metastasis. It is found that some MRI features of primary rectal masses could predict EMVI, postoperative local recurrence and synchronous distant metastasis, some of which could be used as clinical indicators for screening high-risk groups. As a prognostically relevant feature in rectal cancer, NP and CS are correlated with the increased risk of EMVI, synchronous metastasis and recurrence. Moreover, NP was an independent risk factor for EMVI. Therefore, according to our research, it is preoperatively predictable for the occurrence risk of local recurrence and distant metastasis after surgery. Relevant treatment could be provided to the high-risk patients before surgery to improve treatment and prognosis. Additionally, follow up interval for high-risk patients should be appropriately shortened for early detection of local recurrence and distant metastasis.

Vascular endothelial growth factor and β-catenin are highly expressed in the marginal cells of tumors with strong biological activity [15]. Therefore, these tumor cells exhibit active proliferation and rapid growth, which may underlie the formation of the NP profile, while these tumor cells are poorly differentiated, invasive, and easily erode the surrounding blood vessels, nerves, and lymph nodes. Therefore, this may be one of the reasons why NP is an independent risk factor for EMVI. The appearance of NP resulted in the enlargement of tumor volume and area, providing a basis for tumor invasion to the peripheral vessels. Moreover, some NP may originate from tumor tissues that invaded into the vascular lumen at the juncture with rectal wall, or from the tumor thrombus in the vascular root cavity attached to the primary tumor mass with broad base. In addition, the NP may originate from the fusion of tumor deposits (TDs) near the tunica adventitia of the rectum with the primary tumor mass. The origin of TDs had close relationship with the vessels. Consequently, TDs themselves can also invade adjacent vessels and develop distant metastasis.

Rectal cancer can cause a host reaction of desmoplastic reaction (DR) which could evoke to form fibrous tissue around the tumor [16]. In addition, radiotherapy can cause fibrosis in normal tissues and tumor necrosis area. DR is an important component of the tumor microenvironment, as well as an independent prognostic factor for poor prognosis of tumor [17]. In particularly, carcino-associated fibroblasts formed during DR are associated with tumor dedifferentiation which could induce tumor budding and further increase the aggression of the tumor itself. DR is considered as an important factor to promote the aggressive tumor growth in advanced tumor stage, which can promote tumor epithelial transformation to accelerate tumor infiltration. Even more, previous studies reported that DR typing as a prognostic parameter may exceed tumor histological differentiation, vascular invasion and tumor stage [18, 19].

CS in this study included DR, EMVI, lymphangitis carcinomatosa [20] and carcinomatous cords. Approximately 25% of the cord shadows arould the colorectal cancer was caused by inflammatory reactions [21]. In terms of HRT2WI or CET1WI, MRI can effectively distinguish between blood vessels and fibrous cords. However, it is not reliable to distinguish fibrosis with and without tumor cells [22]. In general, CS had close relationship with the biological behavior of the malignancies.

In our study, IN was defined as irregularly shaped nodes which was mainly metastatic lymph nodes and small proportion of TDs. Therefore, it can be considered that IN was an independent predictor of EMVI. In previous studies, local lymph node metastasis and EMVI were risk factors mutually interacted [23, 24]. Since the 20th century, TDs have been recognized as a discrete nodule of tumor in the pericolonic and perirectal adipose tissue or adjacent mesentery without identifiable vascular structure or lymph node [25], which are small tumors in adipose tissues outside the colon or rectum and not in lymph nodes. TDs had been defined as pericolic or perirectal fats with discontinuous tumor spread, extravascular spread with venous invasion or totally replaced lymph nodes by American Joint Committee on Cancer Staging Manual 7th Edition (AJCC 7th TNM) and College of American Pathologist cancer protocol [26, 27]. There was a correlation between the origin of the TDs with venous invasion, lymphatic invasion, nerve sheath infiltration, and continuous growth [28, 29]. The presence of the TDs may also indicate low differentiation and high invasiveness of tumor, while TDs also frequently invaded adjacent blood vessels and caused distant metastasis. Consequently, it is reasonably speculated that IN is correlated with EMVI and synchronous metastasis in rectal cancer. However, the MRI could not effectively differentiate the metastatic lymph nodes and TDs. Therefore, we could not evaluate the metastatic lymph nodes and TDs separately.

EMD was significantly associated with EMVI, synchronous metastasis and local recurrence. Koca et al. reported that the degree of tumor invasion was related with local recurrence after surgery [30], which is consistent with our research findings. Patients with larger EMD and advanced T-stage would present deeper and more aggressive tumor infiltration, together with larger lesion volume and more vessel involvement, as well as high incidence of vascular invasion and synchronous metastasis [31]. Moreover, when the tumor is large, it tends to invade the surrounding organs and rectal fascia which induces difficult complete removal of rectal mass and high recurrence. In this study, we found that compared with local mass, rectal cancer with circumferential infiltrative growth was more likely to show synchronous metastasis and local recurrence which showed similar trends with EMD, MRI T stage and tumor length. The circumferential infiltrative growth pattern resulted in large tumor area and higher chance of exposure to blood vessels, nerves, lymph nodes, which consequently induced higher metastasis rate and local recurrence.

Peritoneal carcinomatosis from colorectal cancer occurs in 20% of patients with non-mucinous colorectal cancer, presenting a synchronous or metachronous sign, of which 6–8% occur only in the peritoneum [32]. In the present study, univariate analysis indicated that PRI was associated with local recurrence of rectal cancer after surgery, and 12 (7.8%) cases of synchronous PRI were detected by MRI. Multivariable analysis indicated that PRI was a crucial risk factor for local recurrence of rectal cancer after surgery. If the primary tumor invades peritoneal reflection, it means that the tumor has penetrated the plasma membrane and the peritoneum has been infiltrated by tumor cells. This may lead to cancer cells implantation or shedding into the abdominopelvic cavity, and it is difficult for surgical eradication. Meanwhile, it is also easy to invade the adjacent blood vessels and lymphatic vessels, which leads to higher tumor recurrence rate and lower survival rate of patients [32].

Many pathological and imaging researchers have found that EMVI is closely correlated with adverse prognostic events such as local recurrence, distant metastasis and tumor-related death of rectal cancer [30–32]. This study also confirmed this result, mEMVI was significantly correlated with synchronous metastasis and local recurrence, and was a risk factor for synchronous metastasis, with an OR value up to 43.7. At present, the detection of EMVI is mainly through postoperative pathology and preoperative MRI, but the detection rate is not the same. A recent study showed that the positive rate of mEMVI was only 21% [33], while in this study, both hEMVI and mEMVI were 32.7%, slightly higher than the results of some studies. This difference may be attributable to variances in sample populations and the use of a combined immunological and elastic fiber staining technique by our pathologists to enhance the detection rate of hEMVI. Furthermore, pairing HRT2WI and CET1WI to assess EMVI may have also contributed to improving the detection rate of mEMVI to some extent, but some recent studies showed that the positive rate of mEMVI could be as high as 51% [34]. The above results were quite different. In this study, NP and mEMVI had a very close value and even a slight advantage in the detection efficiency of EMVI. The sensitivity and AUC were slightly larger than mEMVI, which may be a beneficial supplement to preoperative EMVI detection. Additionally, the nomogram based on tumor morphology exhibits excellent predictive performance, enhancing the detection rate and accuracy of preoperative EMVI. It effectively identifies high-risk patients with EMVI and aids in clinical treatment decisions, maximizing therapeutic benefits for these individuals.

This study has some limitations that must be acknowledged. Firstly, our study’s single-center design and two-year follow-up period resulted in relatively small sample sizes for recurrence (14/153, 9.2%) and synchronous metastasis (16/153, 10.5%), potentially leading to inflated odds ratios and affecting the predictive performance of the nomogram model. Previous study reported that the local recurrence of rectal cancer after a curative resection was in a range of 5.6 to 11.2%[33, 35, 36]. Secondly, the distant metastatic lesions were diagnosed by imaging data and biopsy and were not determined by pathological examination for various reasons, which may result in misclassification. Due to the limitation of MRI imaging solution, we did not evaluate the lymph nodes and TDs separately. Thirdly, due to being a single-center study with a small sample size of synchronous metastasis and recurrences, external validation was not conducted and splitting the samples into training and validation sets for model validation was not feasible. This may have impacted the predictive performance of the model. Fourthly, as a retrospective analysis, it is difficult to accurately compare the MRI and pathological findings. Fifthly, in this study, a small proportion of patients underwent preoperative neoadjuvant therapy, resulting in an extended time interval between MRI and surgical intervention. If patients exhibited a poor response to neoadjuvant therapy, this prolonged interval could lead to tumor progression. Conversely, if the neoadjuvant treatment demonstrated favorable efficacy, it could induce tumor necrosis and fibrosis, thus causing inconsistency between the postoperative pathological status and the baseline tumor status as indicated by preoperative MRI.

## Conclusion

In conclusion, multiple MRI features of tumor morphology are significantly associated with several adverse prognostic indicators, and the nomogram based on NP has excellent predictive performance for preoperative EMVI. mEMVI is a crucial risk factor for synchronous metastasis and local recurrence. PRI is a crucial risk factor for postoperative local recurrence of rectal cancer. These findings facilitate the preoperative selection of patients with high risk of EMVI, postoperative recurrence and distant metastasis based on MRI. For these patients, appropriate treatment strategy and follow-up examination could improve the prognosis as personalized treatment.

## Data Availability

The datasets used and/or analysed during the current study are available from the corresponding author on reasonable request.
